# Role of the dorsal periaqueductal gray in posttraumatic stress disorder: mediation by dopamine and neurokinin

**DOI:** 10.1038/s41398-019-0565-8

**Published:** 2019-09-17

**Authors:** M. L. Brandão, T. A. Lovick

**Affiliations:** 1 Instituto de Neurociências e Comportamento, Avenida do Café, 2450, 14050-220 Ribeirão Preto, SP Brazil; 2 NAP-USP-Neurobiology of Emotions Research Centre (NuPNE), Ribeirão Preto Medical School of the University of São Paulo (FMRP-USP), Av. Bandeirantes, 3900, Ribeirão Preto, São Paulo 14049-900 Brazil; 3 School of Physiology, Pharmacology and Neuroscience, University of Bristol, Bristol, B15 2TT UK

**Keywords:** Learning and memory, Scientific community

## Abstract

In susceptible individuals, exposure to intensely traumatic life events can lead to the development of posttraumatic stress disorder (PTSD), including long-term dysregulation of the contextual processing of aversive stimuli, the overgeneralization of learned fear, and impairments in the ability to learn or respond to safety signals. The neuropathophysiological changes that underlie PTSD remain incompletely understood. Attention has focused on forebrain structures associated with fear processing. Here we consider evidence from human and animal studies that long-lasting changes in functional connectivity between the midbrain periaqueductal gray (dPAG) and amygdala may be one of the precipitating events that contribute to PTSD. Long-lasting neuroplastic changes in the dPAG can persist after a single aversive stimulation and are pharmacologically labile. The early stage (at least up to 24 h post-stimulation) involves neurokinin-1 receptor-mediated events in the PAG and amygdala and is also regulated by dopamine, both of which are mainly involved in transferring ascending aversive information from the dPAG to higher brain structures, mainly the amygdala. Changes in the functional connectivity within the dPAG-amygdala circuit have been reported in PTSD patients. We suggest that further investigations of plasticity and pharmacology of the PAG-amygdala network provide a promising target for understanding pathophysiological circuitry that underlies PTSD in humans and that dopaminergic and neurokininergic drugs may have a potential for the treatment of psychiatric disorders that are associated with a dysfunctional dPAG.

## Introduction

Over the last three decades, dysregulation of neural mechanisms mediating the neurobiology of fear and their association with many types of anxiety has become the subject of considerable debate. Most of this prior work focused on telencephalic structures, connections, and transmitters whereas critical structures in the lower brainstem have only recently begun to receive adequate research attention. The purpose of the present review is to highlight the functional role of the mesencephalic periaqueductal gray (PAG) as a central structure controlling the defensive reactions that are associated with posttraumatic stress disorder (PTSD). In this context dysregulation of the dorsal PAG (dPAG)–amygdala pathway and the potential role of dopamine and neurokinins is considered. We first briefly define PTSD and current thinking regarding its biological basis and then provide an overview of the neuroanatomy of the dPAG, its internal structure, primary transmitters, neural connections, and its potential role in the pathophysiology of PTSD.

## Posttraumatic stress disorder: definition

Posttraumatic stress disorder (PTSD) can develop after experiencing or witnessing a range of traumatic events, such as sexual assault, warfare, traffic collisions, or other threats to a person’s life (e.g., terrorist attack, natural disaster, serious accident, personal assault or abuse, or the sudden death of a loved one)^1^. Sufferers of PTSD experience periods of intense distress, including flashbacks, nightmares, repetitive and distressing images or sensations, physical sensations (e.g., pain, sweating, feeling sick, or trembling), heightened reactivity to external stimuli, anxiety, and depressed mood^1^. This clinical picture can lead the individual to avoid situations that elicit memories of the trauma; they may become very anxious and continuously aware of perceived threats, become easily startled, and have difficulty relaxing. This state of mind, known as hyperarousal, often leads to irritability, angry outbursts, insomnia, and difficulty in concentrating^2^. The core features of PTSD involve intrusive thoughts, memories, and perceptions (flashbacks). These recur long after the initial stress and are often unrelated to the context where the individual currently is, as if the sufferer is re-experiencing the initial traumatic event^2^.

## Biological basis of PTSD

Not every individual who is exposed to a traumatic event experiences PTSD and even in those who do, multiple factors undoubtedly contribute to its development. Trauma can produce extremely long-lasting effects on the nervous system. PTSD patients and those who experience childhood trauma have been shown to present signs of accelerated epigenetic aging^3^. Evidence for a genetic predisposition to develop PTSD is an important determinant of disease progression^4^. The environment in which the trauma was experienced may also be a triggering factor^5,6^.

There is compelling evidence for structural and functional abnormalities within fear-processing regions within the forebrain in patients with PTSD, particularly within the prefrontal cortex (PFC), anterior cingulate, insula, hippocampus and amygdala^7^. Deficits in the extinction of fear memory and the way this impacts on subsequent interpretations of and reactivity to sensory events may be at the core of PTSD. In unaffected (healthy) individuals, the reflex response to a traumatic fear-evoking stimulus (unconditioned response [UR]) normally becomes suppressed over time unless it is reinforced by re-exposure to a similar situation (contextual fear conditioning). Various animal models of PTSD have focused on the extinction of fear, based on the premise that this process is impaired in PTSD^8^. Patients with PTSD continue to exhibit a robust conditioned fear response to a previously extinguished conditioned stimulus [CS]^9^. The use of extinction-based behavioral therapies for the treatment of PTSD is based on this tenet^10–12^.

Individuals who develop PTSD are generally not re-exposed to the original aversive context. Rather, their symptoms appear to be triggered by the retrieval of memories of the initial traumatic event or by cues that represent generalization of the expectation of an aversive outcome. In classical Pavlovian terms, extinction implies gradual waning of the conditioned response (CR) as a consequence of non-reinforcement of the conditioned stimulus (CS). In PTSD, the individual does not experience the CS again. Instead, they appear to generalize the context of the original traumatic event (CS) so that other stimuli trigger an aversive reaction. A key structure involved in mediating fear-evoked behavior is the periaqueductal gray matter, particularly its dorsal part (dPAG). The way in which dPAG processes aversive information following exposure to a traumatic event may be highly relevant to the development of PTSD. In this respect a hypothesis that is gaining currency is that people living with PTSD experience dysregulation of the contextual processing of aversive stimuli, leading to the overgeneralization of learned fear and impairments in the ability to learn or respond to safety signals^13^. Thus, rather than a deficit in fear extinction per se, although this may be a contributory factor, PTSD may represent a fundamental malfunction within the fear-processing circuitry.

Fear is a mental state that is induced by perceived danger or threat, which causes a change in behavior and related autonomic changes that constitute an adaptive response of the organism in order to promote survival^14,15^. Thus, the individual may flee, hide, or freeze to escape from perceived traumatic events. One of the key brain areas that integrates the response to aversive stimuli and elaborates appropriate adaptive behavioral responses is the midbrain periaqueductal gray (PAG)^16^. There are several indications that exposure to an aversive experience can lead to long-lasting plastic changes in the PAG, which may influence subsequent processing of fear-related behaviors.

## Basics of normal PAG function

The PAG usually acts as a hub that integrates a vast array of bodily functions that are directed toward the survival of the individual. It is an essential brain region for controlling cardiovascular and respiratory function, temperature regulation, micturition, vocalization, sexual behavior, and responsiveness to pain. It is a key center for integrating emotional behaviors, such as anxiety, aggression, and defensive reactions when individuals face with a proximal or approaching threat^14–19^.

The PAG is essentially a tubular structure that surrounds the aqueduct. With regard to its role in emotional behavior, it is broadly organized into four longitudinal columns^20–22^. The dorsolateral and dorsomedial columns, commonly referred to as the dorsal PAG (dPAG), are separated from the ventrolateral column (vlPAG) by the lateral column^20–22^. The ventral and dorsal columns appear to be involved in oppositional forms of defensive behavior, namely freezing and escape, respectively^21^. The dPAG is involved in mediating several defensive reactions and is part of the fight/flight system^14,15,17^. Since the early 1980s, the dPAG has been considered the main output center for defensive behaviors that represent an active coping response to a stressful challenge^14,15,18^. This view stems from fight-or-flight reactions accompanied by excitation of the sympathetic nervous system and analgesia that can be evoked by electrical stimulation of the dPAG in animals^14,15,18–21^. That activation of the dPAG produces an aversive experience is reflected by the readiness of animals to learn tasks that will terminate electrical stimulation of the dPAG^23^. In humans too, very unpleasant fear-like sensations are evoked by stimulation of the dPAG^24–26^. Such sensations include feelings of terror or impending death, a desire to flee accompanied by palpitations, blushing of the face and neck, and respiratory arrest or hyperventilation.

In contrast to the dPAG, activation of the ventral PAG (vPAG) appears to be involved in more passive patterns of behavioral responses that are manifested in rats and other species by immobility and inhibition of the sympathetic nervous system activity^22,27^.

**Fig. 1:**
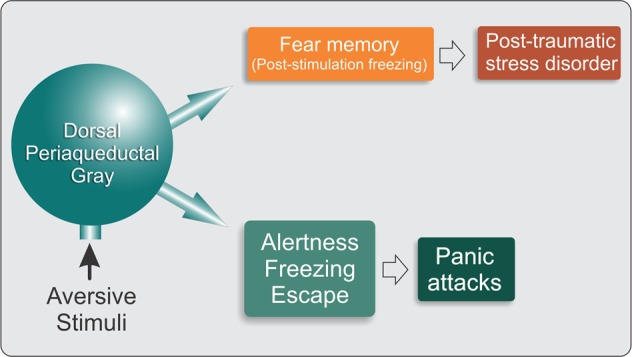
Hypothetical schematic diagram of involvement of the midbrain tectum in fear. Acute fear stimuli activate midbrain tectum structures and produce defensive reactions, characterized by behavioral and autonomic responses that are characteristic of panic attacks. Persistent or intense fear stimuli cause stress, which can lead to the transfer of aversive information to higher brain structures that are associated with panic disorder or posttraumatic stress disorder

In the absence of connections with the forebrain, the midbrain tectum can coordinate an integrated defensive response, first reported more than a century ago as the “sham rage” that could be triggered by nociceptive inputs in decerebrated animals^28^. In the intact brain, forebrain structures impose a level of inhibition on the PAG so that defensive behavior is “released” by the forebrain only as an outcome of the integration and processing of sensory and contextual cues from the environment. The PAG receives substantial sensory input directly from the spinal cord as well as auditory and visual information from the tectum (inferior and superior colliculi)^29^. It is reciprocally connected with the amygdala, as well as other forebrain structures that are involved in processing emotions closely associated with the production of fear responses to proximal danger^14,15,17,30–33^. There is clear evidence that as well as integrating the motor manifestations of fear or defensive behavior the dPAG is involved in assimilating sensory input that may trigger the fear behavior. Neurons in the dPAG are activated by unconditioned fear stimuli (e.g., footshock or exposure of rats to cat odor)^34–36^ and also respond to conditioned stimuli that are associated with aversive events^36^. However, relatively little is known about the functioning of the neurocircuitry that underlies the transfer of aversive information from the dPAG to the higher brain structures and the processing that determines the production of the final motor outputs (Fig. 1). What is certain however, is that the defense reactions that are organized and integrated at the level of the dPAG utilize a multimediated process that involves γ-aminobutyric acid (GABA), serotonin, opioids, excitatory amino acids, nitric oxide, and cannabinoids^12,15,17^.

## Involvement of the PAG in the pathophysiology of PTSD: long-lasting changes

Evidence is accumulating to suggest that changes in the dPAG fear/anxiety circuitry may contribute to the pathology of PTSD. There appears to be remarkable plasticity within this PAG-amygdala circuitry even following a single activation of the aversive system of the PAG.

In rats, a single aversive experience evoked by stimulation of the dPAG produced long-lasting increases in anxiety and fear-like behaviors (assessed in the elevated plus-maze or elevated T-maze) which persisted for at least two weeks following the stimulation^37–39^. Other studies of animals exposed to traumatic stressful challenges which engage the PAG also indicate that it undergoes functional changes that far outlast its initial response to the aversive stimulus. In rats that were exposed to predator stress (a putative animal model of PTSD), persistent neuroplastic changes (expression of the cellular transcription factor phosphorylated cyclic adenosine monophosphate response element binding protein [pCREB]) were detected in the dPAG 7 days following the traumatic event^40^. Another more recent study in rats showed that early-life maternal separation (a model of childhood separation anxiety) decreases the thresholds of panic-like behaviors elicited in response to electrical stimulation of the dPAG when tested in adulthood some months later^41^. This finding is particularly interestingly when viewed in the context of the recent report showing increased sensitivity to panicogenic challenge in patients with PTSD^42^, which is consistent with development of long-lasting trauma-induced changes in the excitability of the panic circuitry within the dPAG.

Several recent imaging studies in humans with PTSD reported changes in the functional activity of PAG circuitry. The main type of PTSD is recognized to be associated with hyperarousal and hyperactivation of the amygdala, whereas the dissociative subtype of PTSD (PTSD+DS) is associated with lower amygdala activation, emotional detachment, and hypoarousal. A recent clinical study employed dynamic causal modeling and found that PTSD was characterized by a pattern of predominant bottom-up connectivity from the PAG to the amygdala and PFC, whereas PTSD+DS patients exhibited predominant top-down connectivity from the PFC to the amygdala and PAG^42^. Thus, “bottom-up” connections from the dPAG to the amygdala and ventromedial PFC predominate in individuals with PTSD compared with normal controls^43^. Another study examined resting-state functional connectivity and found that compared with controls, PTSD patients exhibited extensive increases in functional connectivity of the dlPAG with brain regions (e.g., dorsal anterior cingulate and anterior insula) that are associated with emotional reactivity, defensive action, active coping strategies, and hyperarousal. On the other hand, PTSD+DS patients exhibited increased functional connectivity of the vlPAG with brain regions (i.e., temperoparietal junction) that are associated with passive coping strategies^44^. These findings suggest that in susceptible individuals, the long-lasting disruption or destabilization of dPAG circuitry after acute exposure to a traumatic aversive event may be a factor that underlies the inappropriate expression of fear behavior in individuals with PTSD (Fig. 1). Closer investigation of neuronal processing within the PAG following exposure to an aversive event may, therefore, provide insight into the neuroplastic changes that lead to PTSD.

## Freezing behavior in rodents as an index of PAG functionality

Freezing behavior, operationally defined as the cessation of all body movement except those necessary for breathing, is a natural response of rodents to threatening or fear-inducing situations. In rodents, freezing is one of the most robust and easily quantifiable components of the aversive behavioral response that is coordinated by the dPAG. Studies of freezing evoked by stimulation of the dPAG may be particularly instructive in understanding the role of the PAG in controlling behavior in animals during the post-trauma period.

Three types of freezing are associated with activation of the PAG:

1. *General freezing behavior*. Freezing is the first response to appear with low-intensity stimulation of the dPAG^13,14^. As the stimulation intensity increases and becomes more aversive to the animal, freezing is superseded by an active escape response. The freezing response to low-intensity stimulation of the dPAG does not depend on the functional integrity of the amygdaloid complex^45^ and most likely represents an unconditioned response to a novel threatening stimulus that is coordinated at midbrain level and mediated by activation of descending pathways from the dPAG without any processing by the forebrain.
2. *Contextual freezing*. Contextual freezing occurs when the rat is placed in an environment where it previously experienced an aversive stimulus, often a footshock. It has long been conceptualized that freezing associated with an aversive context requires activation of neurocircuitry that includes the hippocampus, amygdala, and medial PFC, all of which are known to be involved in learning and memory processes that enable context-dependent behaviors^27^. This kind of freezing can also be evoked by stimulation of the ventral PAG (vPAG) which also involves activation of the amygdaloid complex^46–49^ and is thought to be related to a passive defensive posture. The freezing response that is evoked by stimulation of the vPAG is distinct from the response that is evoked by activation of the dPAG. Whilst both engage the same pattern of motor response, lesions of the vPAG do not change freezing or escape thresholds that are associated with electrical stimulation of the dPAG^33,50,51^. Thus, the dPAG and vPAG appear to operate independently.
3. *dPAG-evoked post-stimulation freezing (dPAG-PSF)*. The third type of freezing occurs following the cessation of dPAG stimulation at intensities that evoke escape behavior. This type of freezing contrasts with low-intensity dPAG-evoked freezing, which ceases on termination of the stimulation. dPAG-PSF is resistant to contextual shifts and persists when rats are placed in a new context immediately after exposure to the initial fearful challenge^33,50,51^. Like the contextual conditioned freezing mediated by the vPAG, dPAG-PSF also depends on pathways that relay information via the amygdala^45^. However, the two forms of freezing probably have distinct functional meanings since contextual freezing ceases with the termination of exposure to the context, whereas dPAG-PSF persists even when the animal has been removed from the aversive context, suggesting that animal is maintained in a state of heightened arousal triggered by activation of the PAG.

Viewed in the context of the persistent neuroplastic changes in the functionality of the PAG that can be triggered by exposure to an aversive event both in rats^39^ and in people living with PTSD^43,44^ a study of dPAG-PSF may be particularly instructive. In rats, there appears to be a period of at least 24h following exposure to an aversive situation when the fear-processing circuitry undergoes a functional change^40^. In normal rats, this effect reversed within 7 days. However, it is conceivable that animals maintained in a non-familiar environment in the post-trauma period or rats from strains with a different genetic disposition or even those that suffered early life stress (which has been shown to predispose to PTSD in humans) may experience much longer-lasting or even permanent changes in the fear-processing circuitry. dPAG-PSF may, therefore, serve as a useful model for studying the mechanism of long-lasting after-effects of exposure to traumatic events.

## dPAG-amygdala connections

The dPAG, amygdala and medial hypothalamus are closely associated with the production of fear responses to proximal danger^14,15,17,31^ and have reciprocal anatomical connections^20^. Fear-evoked responses in the amygdala recruit the hypothalamus and PAG as an output fear circuit^17,52^. Importantly, inactivation of the PAG attenuates fear-evoked responses in the amygdala, indicating that the PAG may relay instructive fear signals to the amygdala^52^. In the search for how these structures communicate with each other, several studies have shown that the PAG is downstream of the amygdala, driving motor outputs toward the appropriate defensive response, such as freezing and escape^20–22,53–55^. The PAG and amygdala are known to be essential for both innate and learned fear^55,56^. Evidence indicates that neurons in both structures are responsive to both conditioned and unconditioned stimuli^36,57–60^ and are necessary for producing fear responses^61,62^. Moreover, stimulation of the amygdala or dPAG is both effective unconditioned stimuli (UCS) in fear conditioning procedures^63,64^.

The basolateral amygdala is involved in processing aversive information^65^, whereas the central nucleus of the amygdala functions as a link between the amygdala and brainstem motor regions that mediate conditioned fear responses^66,67^. The amygdala also functions as an interface for processing unconditioned and conditioned fear. The sensorimotor gating system is recruited by traumatic stress and depends on neural activity in the PAG and ascending projections to the amygdala^68,69^. The fact that dPAG-PSF is inhibited by inactivation of the amygdala may be related to the fact that the dPAG integrates sensory information that allows the recognition of threatening stimuli^45^. Indeed, freezing behavior has been proposed to allow animals to acquire aversive information from the environment, which is likely relayed through the laterodorsal nucleus of the thalamus to higher brain structures^70,71^. Fibers that originate from the dPAG innervate various forebrain regions, including the amygdaloid complex, through the medial forebrain bundle^29,32^. Fanselow^48^ and De Oca et al.^49^ suggested that connections between the dPAG and amygdaloid complex may modulate the occurrence of post-encounter freezing behavior. Thus, ascending dPAG efferents to the amygdala appear to be activated during dPAG-PSF so as the PAG continues to transmit such information after the stimulation stops.

The above evidence suggests that the dPAG conveys information about the US to the amygdala, which elaborates both innate and learned fear responses, and also claims a bottom-up control of defensive reactions in the brain. As well as processing aversive information that is encoded in the amygdala, the dPAG is an important relay station for encoded aversive information that is stored in higher brain structures. The medial PFC and anterior cingulate cortex play a crucial role in short-term memory and have also been associated with freezing that is induced by the activation of neural substrates of fear in the dPAG^70,71^. Interference with this system prevents information from staying in memory after the removal of associated environmental cues. Indeed, rats with lesions of the infralimbic cortex that were subjected to an aversive conditioning procedure exhibited significant reductions of conditioned freezing and ultrasonic vocalizations^72^.

**Fig. 2:**
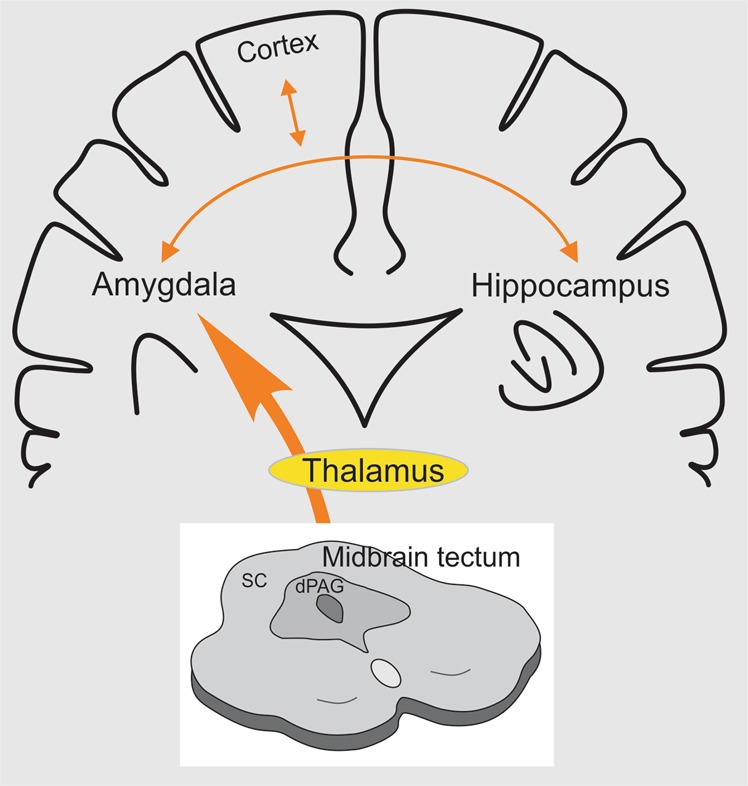
Schematic representation of the connections between midbrain tectum and amygdala, either directly or through the mediodorsal thalamus. Dysfunctional dPAG-amygdala circuitry fed with all sorts of aversive stimuli that arrive at the midbrain tectum in PTSD disrupts the normal functioning of other prosencephalic centers such as the prefrontal cortex and the hippocampus, with which the amygdala has critical neural connections. SC: superior colliculus. dPAG: dorsal periaqueductal gray

Given the evidence of dysfunctional PAG-amygdala circuitry in PTSD, particularly in relation to sensorimotor gating, one question that naturally arises is whether blocking the bridge between the midbrain tectum and higher structures (e.g., the amygdala) would be effective in preventing the development of hypersensitivity following exposure to a traumatic event (Fig. 2). In clinical practice instructing PTSD patients to recall a traumatic memory whilst simultaneously orienting to alternating bilateral sensory stimulation (ABS) such as attending to a swinging light is effective in producing long-lasting attentuation of fear^73^. A comparable effect evoked in mice is associated with long-lasting (weeks) attentuation of excitability of fear-encoding neurons in the amygdala, which could be prevented by optogenetically silencing the path from the midbrain tectum to the amygdala^74^. Whilst optogenetic silencing is not available in humans, the ability to manipulate the PAG-amygdala circuitry pharmacologically may be a potential therapeutic strategy.

## Pharmacological strategies to inhibit the transfer of aversive information from the dPAG to the amygdala following a traumatic event

Below, we present pharmacological evidence that has been obtained with animal models of anxiety designed to examine the chemical mediation of sensorimotor gating that is associated with the PAG and its connections with the amygdala. We have identified two neurotransmitters, dopamine and neurokinin, which appear to be important in the sensorimotor gating of the dPAG^38,39,75,76^. Our recent studies investigated the effects of neurokinin and dopamine agonists or antagonists that were administered either systemically or directly into the midbrain tectum (dPAG and superior and inferior colliculi), where high densities of dopamine D_2_ receptor binding sites have been identified^77^. Thus, the reactivity of neural substrates of fear in the midbrain tectum, measured by auditory-evoked potentials in response to loud sounds, was enhanced by the D_2_ receptor antagonists, sulpiride and haloperidol, that were locally injected into this structure^78^. Supporting the modulatory role of dopaminergic neurons in defensive reactions that are elaborated in the midbrain tectum, we showed that the D_2_ receptor antagonist sulpiride enhanced avoidance behavior (i.e., increased switch-off responses to light presentation as the aversive US) and enhanced fear-like behavior in the open arms of the elevated plus maze when infused in either the superior colliculus or PAG in rats^79^. Recent studies in our laboratory showed that intranasal dopamine application reduced escape behavior in two tests of unconditioned fear (i.e., escape from bright light and ultrasonic vocalization response to immobilization)^80^, attenuated footshock-induced freezing in training, test, and retest sessions in fear extinction conditioning, and reduced PSF following electrical stimulation of the dPAG at the escape threshold^81^. Dopamine neurons in the zona incerta (A13 zone) have been suggested to be the possible source of dopaminergic input that is involved in modulating the neural substrates of fear in the midbrain tectum^82,83^.

In another study, dPAG-PSF was inhibited by injection of the NK1 receptor antagonist spantide into the central nucleus of the amygdala^38,39^. This effect was specific to post-stimulation events since aversive freezing or escape thresholds remained unaffected. Injections of spantide also prevented the aversive effects of electrical stimulation of the dPAG, evaluated in the elevated plus maze 1 day later^38,39^. However, the NK1-mediated effect was labile, and NK1 receptor antagonists failed to block dPAG-PSF tested 7 days later^38^. These findings are particularly prescient in view of recent reports in humans that NK1 receptor availability in the amygdala is associated with anxiety-related personality traits in healthy subjects^84^. NK1 receptor availability correlated positively with trait anxiety but negatively with extraversion consistent with a modulatory role for the SP-NK1 system in human anxiety.

**Fig. 3:**
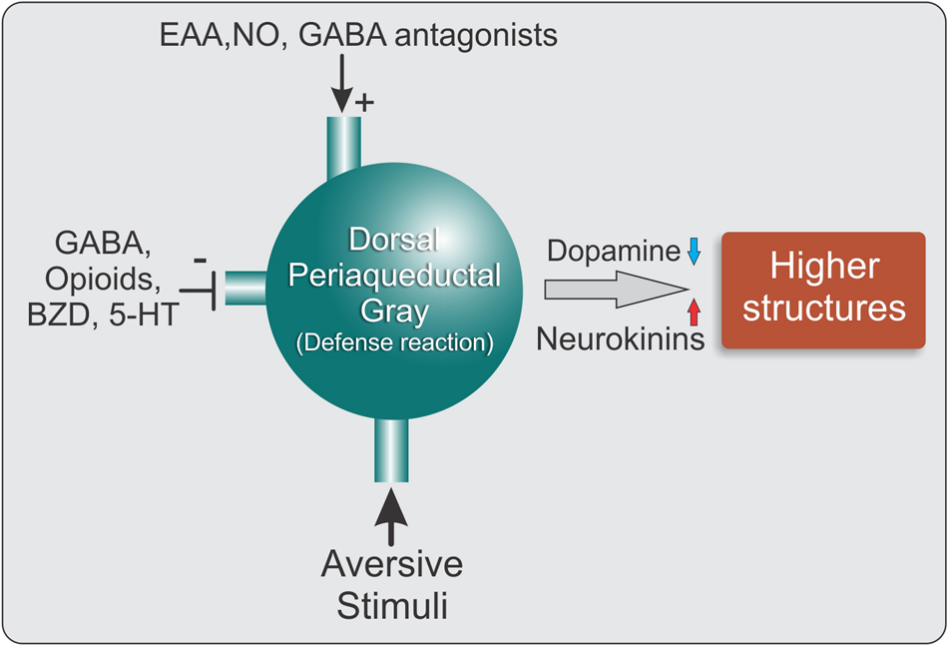
Schematic diagram of neurotransmission in the dPAG in acute and persistent aversive stimulation of the dPAG. Acute fear stimuli activate the dPAG, which produces defense reactions, characterized by behavioral and autonomic responses. The neural substrates of fear in these defense reactions are subjected to excitatory (+) and inhibitory (−) modulatory influences. The passage of aversive information to higher encephalic structures is under the inhibitory control of dopaminergic mechanisms at the midbrain level. NK also mediates the processing of aversive information at the amygdala level. GABA, γ-aminobutyric acid; BZD, benzodiazepines; 5-HT, 5-hydroxytryptamine (serotonin); EAA, excitatory amino acids; NO, nitric oxide

The PAG coordinates behavioral manifestations of fear and aversion and is also an important area that integrates and coordinates the processing of sensory information that is related to fear and aversion. The present review provides evidence of the plasticity of PAG functionality during the period that follows an acute traumatic event and the emergence of a transference process that is confined to bundles of the brainstem (i.e., mesencephalon), culminating in such anxiety disorders as PTSD. These neural mechanisms of aversion and intense fear at the level of the dPAG are normally contained by tonic and inhibitory processes that are mediated by various neurotransmitters, especially GABA. Panic or PTSD may occur when these inhibitory mechanisms are weakened by traumatic stress. Thus, a neural network that comprises projection neurons and interneurons and their inhibitory mediators acts on the dPAG to regulate alterations of emotional behavior that result from the activation of fear substrates in this region. Another disturbance involves the dysregulation of mechanisms that prevent the passage of this information from the mesencephalic PAG to higher structures (e.g., amygdala) that serve as relays to other aversive information storage areas, such as the hippocampus and cerebral cortex. Inhibitory mechanisms must be engaged in the dPAG to prevent the transmission of aversive information to neural fields that modulate cognition. The weakening of such inhibitory mechanisms may result in the development of PTSD. Pharmacological tools may be beneficial for restoring adequate nhibitory/containment mechanisms within this network to interrupt this transference process that can result in PTSD. Dopaminergic drugs have been proposed to serve as a brake to transference mechanisms that relay information that is related to past traumatic experiences. The substance P-NK1 system appears to play an opposing role in this process. Dysfunctional dopaminergic and substance P-NK1 systems in the dPAG-amygdala circuit may be a promising therapeutic target for the treatment of PTSD (Fig. 3).

## References

[1] American Psychiatric Association. (2013). Diagnostic and Statistical Manual of Mental Disorders.

[2] Fenster RJ, Lebois LAM, Ressler KJ, Suh J (2018). Brain circuit dysfunction in posttraumatic stress disorder: from mouse to man. Nat. Rev. Neurosci..

[3] Wolf EJ (2018). Traumatic stress and accelerated DNA methylation age: a meta-analysis. Psychoneuroendocrinology.

[4] Duncan LE, Cooper BN, Shen H (2018). Robust findings from 25 years of PSTD genetics research. Curr. Psychiatry Rep..

[5] Stein MB, Rothbaum BO (2018). 175 years of progress in PTSD therapeutics: learning from the past. Am. J. Psychiatry.

[6] Stevens JS, Jovanovic T (2018). Role of social cognition in post-traumatic stress disorder: a review and meta-analysis. Genes Brain Behav..

[7] Pitman RK (2012). Biological studies of post-traumatic stress disorder. Nat. Rev. Neurosci..

[8] Singewald Nicolas, Holmes Andrew (2018). Rodent models of impaired fear extinction. Psychopharmacology.

[9] Milad MR (2009). Neurobiological basis of failure to recall extinction memory in posttraumatic stress disorder. Biol. Psychiatry.

[10] Milad MR, Quirk GJ (2012). Fear extinction as a model for translational neuroscience: ten years of progress. Annu. Rev. Psychol..

[11] Rauch SL, Shin LM, Phelps EA (2006). Neurocircuitry models of posttraumatic stress disorder and extinction: human neuroimaging research—past, present, and future. Biol. Psychiatry.

[12] Ressler KJ, Mayberg HS (2007). Targeting abnormal neural circuits in mood and anxiety disorders: from the laboratory to the clinic. Nat. Neurosci..

[13] Lisieski MJ, Eagle AL, Conti AC, Liberzon I, Perrine S (2018). A Single-prolonged stress: a review of two decades of progress in a rodent model of post-traumatic stress disorder. Front. Psychiatry.

[14] Brandão ML, Anseloni VZ, Pandóssio JE, De Araújo JE, Castilho VM (1999). Neurochemical mechanisms of the defensive behavior in the dorsal midbrain. Neurosci. Biobehav. Rev..

[15] Brandão ML (2005). GABAergic regulation of the neural organization of fear in the midbrian tectum. Neurosci. Biobehav. Rev..

[16] Deakin JFW, Graeff FG (1991). 5-HT and mechanisms of defense. J. Psychopharmacol..

[17] Graeff, F. G. in *The Neurobiology of Anxiety* (series title: Handbook of Anxiety, vol. 3) (eds. Burrows G. D., Roth M. & Noyes R.) 307–354 (New York, Elsevier, 1990).

[18] Lovick TA (1993). Integrated activity of cardiovascular and pain regulatory systems: role in adaptive behavioural responses. Prog. Neurobiol..

[19] Lovick TA (2000). Panic disorder: a malfunction of multiple transmitter control systems within the midbrain periaqueductal gray matter?. Neuroscientist.

[20] Bandler, R., Carrive, P., Depaulis, A. in *The Midbrain Periaqueductal Gray Matter: Functional, Anatomical, and Neurochemical Organization*(eds. Depaulis, A. & Bandler, R.) 1–8 (New York, Plenum, 1991).

[21] Bandler R, Shipley MT (1994). Columnar organization in the midbrain periaqueductal gray: modules for emotional expression?. Trends Neurosci..

[22] De Paulis A, Keay KA, Bandler R (1992). Longitudinal neuronal organization of defensive reactions in the midbrain periaqueductal gray region of the rat. Exp. Brain Res.

[23] Schmitt P, Eclancher F, Karli P (1974). Etude des systèmes de renforcement négatif et de renforcement positif au niveau de la substance grise centrale chez le rat. Physiol. Behav..

[24] Nashold BS, Wilson WP, Slaughter DG (1969). Sensations evoked by stimulation of the midbrain of man. J. Neurosurg..

[25] Amano K (1978). Single neuron analysis of the human midbrain tegmentum: rostral mesencephalic reticulotomy for pain relief. Appl. Neurophysiol..

[26] Young RF (1989). Brain and spinal stimulation: how and to whom!. Clin. Neurosurg..

[27] Maren S, Phan KL, Liberzon I (2013). The contextual brain: implications for fear conditioning, extinction and psychopathology. Nat. Rev. Neurosci..

[28] Woodworth RS, Sherrington CS (1904). A pseudaffective reflex and its spinal path. J. Physiol..

[29] Al-Khater KM, Todd AJ (2009). Collateral projections of neurons in laminae I, III, and IV of rat spinal cord to thalamus, periaqueductal gray matter, and lateral parabrachial area. J. Comp. Neurol..

[30] Cameron AA, Khan IA, Westlund KN, Cliffer KD, Willis WD (1995). The efferent projections of the periaqueductal gray in the rat: a Phaseolus vulgaris–leucoagglutinin study. I. Ascending projections. J. Comp. Neurol..

[31] Brandão ML, Cardoso SH, Melo LL, Motta V, Coimbra NC (1994). Neural substrate of defensive behavior in the midbrain tectum. Neurosci. Biobehav. Rev..

[32] Rizvi TA, Ennis M, Behbehani MM, Shipley MT (1991). Connections between the central nucleus of the amygdala and the midbrain periaqueductal gray: topography and reciprocity. J. Comp. Neurol..

[33] Vianna DML, Brandão ML (2003). Anatomical connections of the periaqueductal gray: specific neural substrates for different kinds of fear. Braz. J. Med. Biol. Res..

[34] Tovote P, Fadok JP, Lüthi A (2015). Neuronal circuits for fear and anxiety. Nat. Rev. Neurosci..

[35] Tovote P (2016). Midbrain circuits for defensive behaviour. Nature.

[36] Watson TC, Cerminara NL, Lumb BM, Apps R (2016). Neural correlates of fear in the periaqueductal gray. J. Neurosci..

[37] De Almeida LP (2006). Prior electrical stimulation of dorsal periaqueductal grey matter or deep layers of the superior colliculus sensitizes rats to anxiety-like behaviors in the elevated T-maze test. Behav. Brain Res..

[38] Carvalho MC, Santos JM, Brandão ML (2015). Dorsal periaqueductal gray post-stimulation freezing is counteracted by neurokinin-1 receptor antagonism in the central nucleus of the amygdala in rats. Neurobiol. Learn. Mem..

[39] Carvalho MC, Veloni AC, Genaro K, Brandão ML (2018). Behavioral sensitization induced by dorsal periaqueductal gray electrical stimulation is counteracted by NK1 receptor antagonism in the ventral hippocampus and central nucleus of the amygdala. Neurobiol. Learn. Mem..

[40] Adamec R, Hebert M, Blundell J (2011). Long lasting effects of predator stress on pCREB expression in brain regions involved in fearful and anxious behavior. Behav. Brain Res..

[41] Borges-Aguiar AC, Schauffer LZ, de Kloet ER, Schenberg LC (2018). Daily maternal separations during stress hyporesponsive period decrease the thresholds of panic-like behaviors to electrical stimulation of the dorsal periaqueductal gray of the adult rat. Behav. Brain Res..

[42] Kellner M (2018). Effects of 35% carbon dioxide (CO_2_) inhalation in patients with posttraumatic stress disorder (PTSD): a double-blind, randomized, placebo-controlled, cross-over trial. J. Psychiatr. Res..

[43] Nicholson AA (2017). Dynamic causal modeling in PTSD and its dissociative subtype: bottom-up versus top-down processing within fear and emotion regulation circuitry. Hum. Brain Mapp..

[44] Harricharan S (2016). fMRI functional connectivity of the periaqueductal gray in PTSD and its dissociative subtype. Brain Behav..

[45] Martinez RC, de Oliveira AR, Brandão ML (2006). Conditioned and unconditioned fear organized in the periaqueductal gray are differentially sensitive to injections of muscimol into amygdaloid nuclei. Neurobiol. Learn. Mem..

[46] Davis M, Raiunnie D, Cassell M (1994). Neurotransmission in the rat amygdala related to fear and anxiety. Trends Neurosci..

[47] Fanselow, M. S. in *The Midbrain Periaqueductal Gray Matter* (eds. Depaulis, A. & Bandler, R.) 151–173 (New York, Plenum, 1991).

[48] Fanselow MS (1994). Neural organization of the defensive behavior system responsible for fear. Psychonomic Bull. Rev..

[49] De Oca BM, De Cola JP, Maren S, Fanselow MS (1998). Distinct regions of the periaqueductal gray are involved in the acquisition and expression of defensive responses. J. Neurosci..

[50] Vianna DML, Graeff FG, Brandão ML, Landeira-Fernandez J (2001). Defensive freezing evoked by electrical stimulation of the periaqueductal gray: comparison between dorsolateral and ventrolateral regions. Neuroreport.

[51] Vianna DML, Graeff FG, Landeira-Fernandez J, Brandão ML (2001). Lesion of the ventral periaqueductal gray reduces conditioned fear but does not change freezing induced by stimulation of the dorsal periaqueductal gray. Learn. Mem..

[52] Johansen JP, Tarpley JW, LeDoux JE, Blair HT (2010). Neural substrates for expectation modulated fear learning in the amygdala and periaqueductal gray. Nat. Neurosci..

[53] Gray TS, Magnuson DJ (1992). Peptide immunoreactive neurons in the amygdala and the bed nucleus of the stria terminalis project to the midbrain central gray in the rat. Peptides.

[54] Lavond DG, Kim JJ, Thompson RF (1993). Mammalian brain substrates of aversive classical conditioning. Annu. Rev. Psychol..

[55] Kim JJ, Jung MW (2006). Neural circuits and mechanisms involved in Pavlovian fear conditioning: a critical review. Neurosci. Biobehav. Rev..

[56] Kim EJ (2013). Dorsal periaqueductal gray-amygdala pathway conveys both innate and learned fear responses in rats. Proc. Natl Acad. Sci. USA.

[57] Back FP, Carobrez AP (2018). Periaqueductal gray glutamatergic, cannabinoid and vanilloid receptor interplay in defensive behavior and aversive memory formation. Neuropharmacology.

[58] Barot SK, Chung A, Kim JJ, Bernstein IL (2009). Functional imaging of stimulus convergence in amygdalar neurons during Pavlovian fear conditioning. PLoS ONE.

[59] Motta SC, Carobrez AP, Canteras NS (2017). The periaqueductal gray and primal emotional processing critical to influence complex defensive responses, fear learning and reward seeking. Neurosci. Biobehav. Rev..

[60] Pascoe JP, Kapp BS (1985). Electrophysiological characteristics of amygdaloid central nucleus neurons during Pavlovian fear conditioning in the rabbit. Behav. Brain Res..

[61] Choi JS, Kim JJ (2010). Amygdala regulates risk of predation in rats foraging in a dynamic fear environment. Proc. Natl Acad. Sci. USA.

[62] LeDoux JE (2012). Rethinking the emotional brain. Neuron.

[63] Di Scala G, Mana MJ, Jacobs WJ, Phillips AG (1987). Evidence of Pavlovian conditioned fear following electrical stimulation of the periaqueductal grey in the rat. Physiol. Behav..

[64] Castilho VM, Macedo CE, Brandão ML (2002). Role of benzodiazepine and serotonergic mechanisms in conditioned freezing and antinociception using electrical stimulation of the dorsal periaqueductal gray as unconditioned stimulus in rats. Psychopharmacology.

[65] Maren S (2008). Pavlovian fear conditioning as a behavioral assay for hippocampus and amygdala function: cautions and caveats. Eur. J. Neurosci..

[66] Amaral DG, Price J (1984). Amygdala-cortical projections in the monkey (*Macaca fascicularis*). J. Comp. Neurol..

[67] Nader K, Majidishad P, Amorapanth P, LeDoux JE (2000). Damage to the lateral and central, but not other, amygdaloid nuclei prevents the acquisition of auditory fear conditioning. Learn. Mem..

[68] Brandão ML, Troncoso AC, de Souza Silva MA, Huston JP (2003). The relevance of neuronal substrates of defense in the midbrain tectum to anxiety and stress: empirical and conceptual considerations. Eur. J. Pharmacol..

[69] Brandão ML, Zanoveli JM, Ruiz-Martinez RC, Oliveira LC, Landeira-Fernandez J (2008). Different patterns of freezing behavior organized in the periaqueductal gray of rats: association with different types of anxiety. Behav. Brain Res..

[70] Ferreira-Netto C, Borelli KG, Brandão ML (2005). Neural segregation of Fos-protein distribution in the brain following freezing and escape behaviors induced by injections of either glutamate or NMDA into the dorsal periaqueductal gray of rats. Brain Res..

[71] Borelli KG, Ferreira-Netto C, Coimbra NC, Brandao ML (2005). Fos-like immunoreactivity in the brain associated with freezing or escape induced by inhibition of either glutamic acid decarboxylase or GABAA receptors in the dorsal periaqueductal gray. Brain Res..

[72] Frysztak RJ, Neafsey EJ (1991). The effect of medial frontal cortex lesions on respiration, “freezing,” and ultrasonic vocalizations during conditioned emotional responses in rats. Cereb. Cortex.

[73] Wurtz H (2016). Preventing long-lasting fear recovery using bilateral alternating sensory stimulation: a translational study. Neuroscience.

[74] Baek J (2019). Neural circuits underlying a psychotherapeutic regimen for fear disorders. Nature.

[75] Brandão ML (2015). Dual role of dopamine D2-like receptors in the mediation of conditioned and unconditioned fear. FEBS Lett..

[76] Brandão Marcus L., Coimbra Norberto C. (2019). Understanding the role of dopamine in conditioned and unconditioned fear. Reviews in the Neurosciences.

[77] Hurd YL, Suzuki M, Sedvall GC (2001). D1 and D2 dopamine receptor mRNA expression in whole hemisphere sections of the human brain. J. Chem. Neuroanat..

[78] Muthuraju S, Nobre MJ, Saito VMN, Brandão ML (2014). Distinct effects of haloperidol in the mediation of conditioned fear in the mesolimbic system and processing of unconditioned aversive information in the inferior colliculus. Neuroscience.

[79] Muthuraju S, Talbot T, Brandão ML (2016). Dopamine D2 receptors regulate unconditioned fear in deep layers of the superior colliculus and dorsal periaqueductal gray. Behav. Brain Res..

[80] Talbot T, Mattern C, Silva MAS, Brandão ML (2017). Intranasal administration of dopamine attenuates unconditioned fear in that it reduces restraint-induced ultrasound vocalizations and escape from bright light. J. Psychopharmacology..

[81] Carvalho, M. C. et al. Intranasal dopamine attenuates unconditioned and conditioned fear related behaviors induced by intramesencephalic and peripheral aversive stimuli. *J. Psychopharmacol*. 10.1177/0269881119862527 (2019).

[82] Bolton AD (2015). A diencephalic dopamine source provides input to the superior colliculus, where D1 and D2 receptors segregate to distinct functional zones. Cell Rep..

[83] Essig J, Felsen G (2016). Warning! Dopaminergic modulation of the superior colliculus. Trends Neurosci..

[84] Hoppe JM (2018). Association between amygdala neurokinin-1 receptor availability and anxiety-related personality traits. Transl. Psychiatry.

